# Impact of a defined bacterial community including and excluding *Megamonas hypermegale* on broiler cecal microbiota and resistance to *Salmonella* infection

**DOI:** 10.1128/aem.00948-25

**Published:** 2025-08-19

**Authors:** Camila Schultz Marcolla, Tingting Ju, Kimberlee Ten, Usha Sivakumar Sharma, Leakhena Moeun, Benjamin P. Willing

**Affiliations:** 1 Department of Agricultural, Food and Nutritional Science, Faculty of Agricultural, Life and Environmental Sciences, University of Alberta 461698, Edmonton, Alberta, Canada; 2 Department of Animal Sciences, Purdue University 123986 , West Lafayette, Indiana, USA; INRS Armand-Frappier Sante Biotechnologie Research Centre, Laval, Quebec, Canada

**Keywords:** broiler, cecal microbiota, defined microbial communities, commensal bacteria, *Salmonella*, microbial transplantation

## Abstract

Intensive broiler production practices impair the transmission of commensal microbes from hens to offspring, resulting in a lower abundance of non-spore-forming strict anaerobic bacteria. We evaluated the effects of introducing a defined community (DC) of bacteria including and excluding *Megamonas hypermegale* to chicks prior to a *Salmonella* challenge. Inoculation with DC resulted in higher phylogenetic diversity (PD) and the dominance of Bacteroidetes species in the cecal microbiota, with a decrease in the relative abundance of *Salmonella* and *Escherichia/Shigella*, as well as a lower *Enterobacteriaceae* load. Substantial shifts in microbiota composition were coupled with subtle changes in metabolites and host responses, including changes in interferon-γ, macrophage colony-stimulating factor, propionate, valerate, and isovalerate concentrations in the ceca. We identified bacterial isolates included in the DC that were able to establish and persist after a single exposure, many of which were members of Bacteroidetes phyla. Although co-culture with *M. hypermegale* was observed to reduce *Salmonella* counts *in vitro*, *in vivo* inoculation of *M. hypermegale* without an accompanying DC was shown to increase splenic *Salmonella* counts. Inoculation with DC containing *M. hypermegale* increased cecal concentration of valerate and propionate. Our findings provide further insight into how DC might be selected to impact infection resistance. The comparison of DC differing by one single isolate is useful to aid our understanding on the role of individual strains in the broiler cecal community, guiding the formulation of DCs that can potentially favor poultry health and production.

**IMPORTANCE:**

Intensive production practices can reduce beneficial gut bacteria in broiler chickens, potentially leading to higher disease risk. We investigated whether introducing a defined community (DC) of commensal bacteria, with or without *M. hypermegale*, could improve gut health and resistance to *Salmonella* in broiler chicks. Our findings show that DC increases microbial diversity and reduces the relative abundance of potential pathogens, like *Salmonella* and *Escherichia/Shigella* in the ceca, which was coupled with subtle changes in the immune responses of the birds and higher concentration of volatile fatty acids in the ceca. This study suggests that inoculation with DC in early-life can alter microbiota composition, providing a potential strategy to be employed in broiler production. However, further research is needed to understand the role of individual bacteria and refine these bacterial communities for practical use in farming, thus enabling the development of natural methods to enhance poultry health and safety.

## INTRODUCTION

Current poultry production practices aim to minimize bird exposure to pathogens that can cause disease and contaminate food products; however, these practices may impair the colonization of the chicken gastrointestinal tract with host-adapted commensal bacteria that have co-evolved with chickens in nature (1–4). In fact, the cecal microbiota of broilers reared in intensive systems was shown to be less diverse and depleted of non-spore-forming strict anaerobic bacteria, especially from the phylum Bacteroidetes, compared to that of age-matched broilers from extensive systems (2). The cecal microbiota of intensively raised broilers was shown to lack bacterial species that are present in extensively raised birds, including members of *Olsenella*, *Alistipes*, *Phocaeicola, Bacteroides*, *Barnesiella*, *Parabacteroides, Megamonas*, and *Parasutterella* genera (2, 5–8). It was also demonstrated that chicks inoculated with cecal contents and bacterial cultures derived from cecal contents were consistently colonized by *Alistipes*, *Phocaeicola, Bacteroides*, *Barnesiella*, *Mediterranea*, *Megamonas*, *Parabacteroides*, *Phascolarctobacterium*, and *Subdoligranulum*, indicating that these bacteria are highly adapted and able to colonize the chicken gut after a single exposure in early-life (9).

Species from the *Megamonas* genus, including *Megamonas rupellensis*, *Megamonas funiformis*, and *Megamonas hypermegale*, have been isolated from chickens (10–12) and were shown to be enriched in the gut microbiota of wild and free-range compared to intensively raised and domesticated birds (2, 5, 6, 13–15). *M. hypermegale* is an anaerobic, gram-negative, non-spore-forming, and non-motile rod that was first isolated from turkey feces (16). It was shown to produce acetic, propionic, lactic, and trace amounts of succinic acids in broth culture (16, 17). Metagenomic analysis indicated that *M. hypermegale* can metabolize hydrogen, potentially reducing the accumulation of H_2_ that can hinder short-chain fatty acid production within the gut (18). *M. hypermegale* has also been associated with *Salmonella* inhibition. One study reported reduction of *in vitro* growth of *Salmonella* Typhimurium by cross-streaking with *M. hypermegale;* however, no inhibition of *Salmonella* load in chicks inoculated with *M. hypermegale* at hatch and challenged with *S*. Typhimurium was observed (17). Chicks inoculated with a defined microbial community containing *Phocaeicola vulgatus* (formerly *Bacteroides vulgatus*), *M. hypermegale*, and 46 other bacterial isolates demonstrated higher resistance to *S*. Typhimurium infection (19). Notably, when the same microbial community was introduced, but *P. vulgatus* and *M. hypermegale* were undetectable in the chick microbiota, no inhibitory effect on *Salmonella* was observed (19). This pattern suggests that *P. vulgatus* and *M. hypermegale* may play key roles in promoting host resistance to *Salmonella* infection (19). *M. hypermegale* abundance has also been negatively associated with *Campylobacter jejuni* abundance in the turkey gut (20). Broilers receiving cecal microbiota transplant showed an increase in *M. hypermegale* abundance associated with reduced severity of necrotic enteritis symptoms (21).

Given the collective evidence suggesting the importance of *M. hypermegale* as a core member of the poultry gut microbiota and its potential ability to reduce pathogen load, we hypothesized that this commensal would effectively engraft and affect bird physiology and disease resistance once introduced to newly hatched chicks. The current study aimed to evaluate the effect of early-life introduction of *M. hypermegale* alone or in combination with a defined community (DC) of bacteria on broiler gut microbiota development, host immune responses, and ability to resist *Salmonella* infection.

## MATERIALS AND METHODS

### Preparation of microbial inocula

Cecal digesta were collected from broilers and layers raised on commercial farms in Alberta, Canada, to isolate commensal bacteria. The culturing, isolation, identification, and sequencing procedures were previously described (2). Strains included in the DC were selected based on various criteria, including ease of culturing in the lab, abundance of similar taxa within the broiler cecal microbiota, ability to colonize the chicken gut, similarity to taxa differing between intensively and extensively raised broilers, reported anti-*Salmonella* activity (Table 1), and carbohydrate utilization capability (Fig. S1).

**TABLE 1:** Characteristics of isolates included in the defined community (DC)^*a*^

ID	Scientific name	% identity	CFU/mL	Criteria
IS1	*Megamonas hypermegale*	99.93	5 × 10^5^	Reduced abundance in intensively raised broilers (2, 5); reported anti-salmonella activity (19); effectively colonize the ceca after a single exposure (9)
B17	*Barnesiella viscericola*	98.09	3.8 × 10^5^	High abundance in chickens exposed to microbial inocula (7, 9, 22); depleted in broilers in intensive systems (2, 6)
C79	*Fournierella massiliensis*	98.39	1.3 × 10^6^	Cultured representative of Ruminococacceae family (2)
IS19	*Phocaeicola vulgatus*	100	1.0 × 10^7^	Reported anti-salmonella activity (19)
IS22	*Bacteroides mediterraneensis*	99.33	9.0 × 10^3^	Likely to be a chicken-adapted species (23)
IS61	*[Ruminococcus] torques*	95.28	<100	Common cultured representative of Lachnospiraceae family (2)
P14B	*Alistipes finegoldii*	99.76	9.0 × 10^4^	Common cultured representative of Rikenellaceae family (2)
PYG19	*Bacteroides gallinaceum*	92.68	2 × 10^7^	Initially isolated from the ceca of an Indonesian chicken (24)
R21	*Subdoligranulum variabile*	99.09	9.0 × 10^3^	Cultured representative of Ruminococacceae family (2)
R23	*Caecibacteroides pullorum*	93.98	2.3 × 10^5^	Absence of AMR (2)
IS108	*Lactobacillus crispatus*	99.81	1.7 × 10^6^	Strains of *L. crispatus* had shown anti-salmonella activity (25)
P14A	*Ligilactobacillus agilis*	99.66	2.6 × 10^9^	Strains of *L. agilis* can be motile and contain flagellin (26)
PYG53	*Ligilactobacillus aviarius*	99.86	2.8 × 10^6^	Strains of *L. aviarius* are strict anaerobes (27), the used strain has no AMR (2)

^*a*^ The scientific names of isolates were assigned based on the highest max score from BLASTn alignment of 16S rRNA sequences against the rRNA/ITS database, with the corresponding percent identity listed. Colony-forming units (CFU/mL) were determined after culturing and enumerating glycerol stocks post one freeze-thaw cycle. The “criteria” column summarizes the rationale for including each isolate in the DC, based on its abundance, functional characteristics, or relevance to chicken microbiota. Additional information is available in Table S1.

Taxonomy assignment of isolates was performed by aligning full-length 16S rRNA sequences of each isolate against the BLAST rRNA/ITS database and comparing genomic DNA to reference genomes (RefSeq Genome Database) using OrthoANIu algorithm as previously described (1). The nearest matches to the isolates included in the DC community based on percentage identity indicated by BLAST were *Alistipes finegoldii* (99.8%), *Bacteroides meditarraneensis* (99.3%), *P. vulgatus* (100%), *Fournierella massiliensis *(98.4%)*, Ligilactobacillus agilis *(99.7%), *Ligilactobacillus aviaries *(99.9%), *Lactobacillus crispatus *(99.8%), *Subdoligranulum variabile *(99.1%)*,* [*Ruminococcus] torques *(95.3%)*, Bacteroides gallinaceum *(92.7%)*, Caecibacteroides pullorum* (previously *Bacteroides uniformis*) (94.0%), *Barnesiella viscericola* (98.1%), and *M. hypermegale* (99.9%) (Table S1). Carbohydrate utilization capability was determined by alignment of genomic DNA sequences against Carbohydrate Active Enzymes Database (CAZyme) (28).

Isolates were cultured on fastidious anaerobe (FA) agar (Neogen, USA), except for *Lactobacillus* and *Ligilactobacillus* species, which were cultured on de Man Rogosa and Sharpe (MRS) agar (BD Difco, USA). Cultures were incubated anaerobically (5% CO₂, 5% H₂, 90% N₂) in an anaerobic chamber (Bactron300, Sheldon Manufacturing, USA) at 37°C for 48 h. A single colony from the agar plate of each strain was picked and inoculated into either FA broth or MRS broth, based on the strain being cultured, and incubated at 37°C for 48 h. Following incubation, individual broth cultures were mixed with liquid casein yeast (LCY) media supplemented with 50% glycerol and 0.05% L-cysteine in a 1:1 ratio. The mixtures were aliquoted into 1.5 mL tubes to create glycerol stocks and stored at −80°C. Cell count and viability of each strain were determined by culturing and enumeration of glycerol stocks after storage (Table S1).

In experiment 1, DC and DC + Mega inocula were assembled under anaerobic atmospheric condition (5% CO₂, 5% H₂, 90% N₂) 2 h prior to inoculation. Glycerol stocks of each isolate were thawed on ice, pooled in equal volumes, and aliquoted into 2 mL tubes. Each aliquot was used to inoculate all birds in a single cage, minimizing oxygen exposure before inoculation. As results indicated that *M. hypermegale* failed to colonize the birds in experiment 1, in subsequent experiments, fresh 24 h *M*. *hypermegale* FA broth cultures were used instead of frozen *M. hypermegale* glycerol stocks.

*Salmonella enterica* serovar Enteriditis SGSC 4901 (PT4) and *Salmonella enterica* serovar Typhimurium ATCC SL1344 were streaked on fresh xylose-lysine-deoxycholate agar (XLD) (Thermo Scientific) and incubated for 18 h at 37°C. A single colony from each plate was selected, inoculated in 5 mL FA broth, and incubated for 18 h at 37°C. After incubation, broth cultures were serial diluted in sterile 1× phosphate-buffered saline, which were subsequently plated onto XLD plates and incubated for 18 h at 37°C for bacterial enumeration. The broths were diluted to achieve 1 × 10^8^ CFU/mL and inoculated to birds via oral gavage at day 5.

### Acids, bile, and oxygen tolerance of *M. hypermegale* and Salmonella inhibition *in vitro*

To evaluate the tolerance of *M. hypermegale* to acids, bile and oxygen, *M. hypermegale* was seeded into 5 mL of FA broth and incubated anaerobically at 37°C for 48 h. For acid tolerance assay, 500 µL of the seeded broth was inoculated into 4.5 mL of FA broth adjusted to pH 2, 3, 5, and 7 using hydrochloric acid. For bile tolerance assay, 500 µL of the seeded broth was inoculated into FA broth containing 0.3%, 0.6%, and 1.2% of sterile porcine bile. All samples were incubated anaerobically at 37°C for 3 h determined based on the average transit time of digesta through the chicken gizzard and small intestine (29). For the oxygen tolerance assay, 500 µL of the seeded broth was inoculated into 4.5 mL of FA broth and incubated aerobically at 37°C for 15, 30, and 60 min. A control sample not exposed to oxygen was considered time 0. After treatments, samples were plated on FA agar and incubated anaerobically at 37°C for 48 h.

To investigate the ability of *M. hypermegale* to inhibit *S*. Typhimurium and *S*. Enteritidis, *in vitro* broth cultures containing 10^4^ CFU/mL of *Salmonella* and 10^4^ CFU/mL *M*. *hypermegale* were co-inoculated into FA broth, while FA broth inoculated with *Salmonella* alone served as the control. Samples were incubated anaerobically at 37°C for up to 48 h and then plated on XLD agar for *Salmonella* enumeration. The inhibition effect was assessed by comparing the *Salmonella* load in the co-cultures to that in control samples. The inhibitory effect of *M. hypermegale* on *S*. Typhimurium was also tested using the “agar slab method” (30). Briefly, broth containing 10^4^ CFU/mL of *M. hypermegale* was spread onto FA agar, incubated anaerobically at 37°C for 48 h, and agar slabs measuring 9 mm in diameter were cut and placed onto a FA plate spread with *S*. Typhimurium, which was further incubated at 37°C for 24 h to measure the zone of inhibition.

### Animal housing and study design

Day-old broilers (Ross 708, Aviagen, Huntsville, AL) obtained from a commercial hatchery were weighed, tagged with individual IDs, and randomly distributed into individually ventilated cages (GR1800 double decker Sealsafe plus, Tecniplast, CA) lined with sterile aspen shavings. Three chicks were housed in each cage with *ad libitum* access to water and food (Laboratory Chick Diet S-G 5065, LabDiet, MO, USA) throughout the experiment. Cages were changed every 3 days and 50 g of bedding materials from the dirty cage were transferred to clean cages to promote exposures to seeded microorganisms. All procedures were performed in a biosafety cabinet. Cages were kept in a temperature-controlled room in a biosafety level 2 facility, with a daily lighting schedule of 12 h light. Room temperature was kept at 30°C for the first 3 days of age and then gradually reduced to 24°C as birds aged. At the beginning of each experiment, 10 chicks were euthanized at arrival, and cecal samples were collected and plated on XLD agar to confirm the absence of *Salmonella* in the baseline microbiota.

In a preliminary experiment (EXP1), 60 day-old chicks weighing 47.24 ± 7.25 g (mean ± standard deviation (SD)) were randomly distributed into cages (3 birds/cage) and allocated into four treatments: Control, Mega, DC, and DC + Mega. Control chicks were inoculated with sterile LCY medium; chicks from the Mega treatment were inoculated with frozen *Μ. hypermegale* glycerol stock containing 5 × 10^5^ CFU/mL; chicks from the DC treatment were inoculated with DC isolates, while chicks from the DC + Mega treatment were inoculated with DC isolates and *M. hypermegale* glycerol stock. All inoculations were performed the day after arrival via oral gavage with 150 µL of inocula. One bird from cage (the lightest one in the cage) was euthanized and sampled at 7 and 14 days old, and the remaining bird was euthanized at 21 days of age.

Results from EXP1 indicated that the *M. hypermegale* isolate used failed to colonize the chicken gut. Thus, a follow-up experiment (EXP2) was designed to test the colonization ability of *M. hypermegale* isolate when provided as a fresh broth culture. Specifically, a total of 48-day-old chicks weighing 44.9 ± 3.8 g (mean ± SD) were randomly distributed into cages and assigned to Control or Mega treatments. Chicks in the Control treatment were inoculated with sterile LCY, while chicks from the Mega treatment were inoculated with 150 µL of fresh *M. hypermegale* broth containing 1 × 10^6^ CFU/mL. Inoculations were performed at the day of arrival and repeated at 48 h after arrival. Two days after the repeated inoculation, chicks in all treatments were infected with *S*. Enteritidis by oral gavage with 1.5 × 10^6^ cells/bird. On day 3 post infection (7 days of age), the lightest chick in each cage was selected for sampling, while the two remaining chicks were sampled 10 days post infection (14 days of age).

Results of EXP2 indicated that the *M. hypermegale* strain used successfully colonized the gut when introduced twice as a fresh broth culture, and a third experiment (EXP3) was conducted using fresh broth culture of *M. hypermegale* in combination with the DC. A total of 72-day-old chicks weighing an average of 46.7 ± 3.7 g (mean ± SD) were randomly distributed into 24 cages (3 birds per cage) and assigned to three treatments: Control, DC, and DC + Mega (8 cages per treatment). The day after arrival, control chicks were inoculated with sterile LCY; chicks in the DC treatment were inoculated with DC isolates; and chicks in the DC + Mega treatment were inoculated with DC isolates and fresh *M. hypermegale* broth (1 × 10^6^ CFU/mL). Inoculations were repeated 24 h later, and 48 h after the second inoculation, all chicks were infected by oral gavage with 150 µL of 1 × 10^7^ CFU/mL *S*. Enteritidis PT4. The lightest chick in each cage was sampled 48 h after infection, whereas the two remaining chicks in each cage were sampled 10 days post-infection (14 days of age).

### Sampling

Chickens were euthanized by cervical dislocation, and the coelomic cavity was opened using aseptic technique. The whole spleen was collected into 1× phosphate-buffered saline (PBS), stored on ice for transport, homogenized by shaking twice at 6.0 m/s for 40 s (FastPrep-24TM 5G, MP Biomedicals), plated on XLD, and incubated for 24 h at 37°C for *Salmonella* enumeration. Cecal digesta and tissues were collected and immediately stored at −80°C. Concentrations of short-chain fatty acids (SCFA) in cecal digesta were determined by gas chromatography as described previously (9). Levels of interferon (IFN)-α, ΙFN-γ, interleukin (IL)−2, IL-6, IL-10, IL-16, IL-21, macrophage colony-stimulating factor (M-CSF), macrophage inflammatory protein (MIP)-1β and MIP-3α, regulated on activation, normal T cell expressed and secreted (RANTES) chemokine, and vascular endothelial growth factor (VEGF) in cecal tissue were determined by a commercial multiplex cytokine assay (Featured—Chicken Cytokine/ Chemokine 12-Plex Assay, Eve Technologies Corporation, CA) as described previously (9). Small intestine segments collected at 0.5 cm proximal and 0.5 cm distal to Meckel’s diverticulum were prepared for histomorphology analysis and stained with hematoxylin eosin as previously described (9). Images of representative cross-sections of small intestine were taken using BioTek Lionheart Imager FX (Agilent Technologies) and analyzed using Agilent Gen 5 software (Gen5 v.3.12).

### *Salmonella, Enterobacteriaceae*, and Bacteroidetes quantification

Quantitative RT-PCR was used to determine total bacteria, *Salmonella*, *Enterobacteriaceae*, and Bacteroidetes load in cecal contents. To generate standards curves, DNA was extracted from pure broth cultures of *S*. Enteritidis and *Alistipes finegoldii* using Wizard Genomic DNA Purification Kit (Promega Corporation, WI, USA) following the manufacturers’ protocol. The reaction mixtures contained 5 µL of SYBR Green SuperMix (Quantabio, US), 0.5 µL of each forward and reverse primer (Table 2), 3 µL of nuclease free water, and 1 µL of DNA diluted to a concentration of 5 ng/µL. The PCR programs consisted of an initial denaturation step of 3 min at 95°C, followed by 40 cycles of 95°C for 10 s and 60°C for 30 s, which was performed on an ABI StepOne real-time System (Applied Biosystems, Foster City, CA).

**TABLE 2:** Assay targets and forward (F) and reverse (R) primers sequences used for qPCR analysis

Assay	Sequence (5′→ 3′)	Reference
Salmonella (enterotoxin gene [stn])	F: CTTTGGTCGTAAAATAAGGCG	(31)
	R: TGCCCAAAGCAGAGAGATTC	
Bacteroidetes (16S rRNA gene)	F: GGARCATGTGGTTTAATTCGATGAT	(32)
	R: AGCTGACGACAACCATGCAG	
*Enterobacteriaceae* (16S rRNA gene)	F: CATTGACGTTACCCGCAGAAGAAGC	(33)
	R: CTCTACGAGACTCAAGCTTGC	
Total bacteria (16S rRNA gene)	F: TCCTACGGGAGGCAGCAGT	(34)

### DNA extraction and 16S rRNA gene amplicon sequencing analysis

Total DNA from cecal digesta was extracted using a QIAamp DNA stool mini kit (Qiagen NV, Netherlands), following the manufacturers’ Pathogen Detection protocol, with minor modifications. Specifically, approximately 100 mg of digesta content was mixed with Inhibitex buffer and 2.0 mm garnet beads (BioSpec Products, Bartlesville, OK), homogenized, and lysed by bead-beating twice at 6.0 m/s for 30 s. The DNA concentrations were determined using Quant-iT Picogreen dsDNA assay kit (Invitrogen, Thermo Fisher Scientific, USA).

Amplicon libraries targeting the V3-V4 regions of the 16S rRNA gene were prepared following the Illumina 16S Metagenomic Sequencing Library Preparation protocol (#15044223 Rev.B). Sequencing was performed on an Illumina MiSeq platform (Illumina Inc, San Diego, CA) using 2 × 300 cycles. Raw sequences were processed using Quantitative Insights into Microbial Ecology 2 v2020.2 (35). Forward and reverse sequences were denoised and truncated at 270 and 220 bp, respectively, and chimeras were removed using DADA2 (v. 2020.2.0) plugin (36). Multiple sequence alignments were performed using MAFFT (37), and phylogenetic trees were generated using the FastTree method (38). Naïve Bayes classifier (39) pretrained on SILVA 138 QIIME compatible database (40) was used for taxonomic classification, and sequences were clustered at 99% identity using majority taxonomy strings. Downstream analyses were performed using phyloseq v.1.40.0 (41), microbiome v.1.18.0 (42), and qiime2R v.0.99.6 (43) packages in R v 1.4.1717 (44). Amplicon sequence variants (ASVs) assigned to Mitochondria family, Chloroplast order, Archaea kingdom, unassigned at the phylum level, and present in less than 10% of the samples or presenting less than 10 reads were removed from the data set. Samples were rarefied at 22,006 reads for downstream analysis. Alpha-diversity was evaluated using phylogenetic diversity and Chao1 indices. Beta-diversity was evaluated using Bray-Curtis distance matrix and visualized by principal coordinates analysis (PCoA). Differentially abundant taxa were identified using DESeq2 with apeglm for logarithmic fold change shrinkage and FDR correction analysis (45, 46). Analysis at the taxon level was performed by merging all the ASVs exhibiting the same taxonomy string using *tax_glom* function (phyloseq package). Spearman correlation analysis was performed using psych v.2.3.3 package (47). Figures were generated using ggplot2 v.3.4.0 (48), GraphPad Prism v.10.4.2, and pheatmap v.1.0.12 (49).

### Colonization ability

Colonization ability of bacterial isolates was determined based on the prevalence of each isolate in the cecal samples of inoculated birds. Specifically, bacteria that were detected in at least half of the inoculated birds were considered prevalent, thus having high colonization ability.

### Statistical analyses

All statistical analyses were performed in R (44). Beta-diversity matrices were analyzed using multivariate homogeneity of group dispersions and permutational multivariate analysis of variance with Benjamin-Hochberg procedure for FDR control using phyloseq v.1.40.0 (41) and vegan v. 2.6-4 (50) packages. Data were tested for normality using the Shapiro-Wilk test and analyzed by one-way ANOVA followed by Tukey’s HSD test if normally distributed, or Kruskal-Wallis and pairwise Dunn test with Bonferroni adjustment for multiple comparisons if distribution was not normal (stats package v. 4.3.3) (44). *Salmonella* translocation in spleen was analyzed using Fisher’s exact test. In all cases, a *P*-value of less than 0.05 was considered statistically significant.

## RESULTS

There were no differences in the body weight among treatments at any of the timepoints measured in all three trials (Table S2). Histological analyses performed on ileal tissues of birds in EXP3 indicated no differences in villus height, crypt depth, villus width, and villus height:crypt depth ratios of birds in control, DC, and DC + Mega treatments (Table S3).

### Salmonella growth was inhibited by co-culturing with *M. hypermegale in vitro*

*M. hypermegale* can endure pH 5, at least 30 min of oxygen exposure, and up to 1.2% bile acid in the media. There was no difference in viable cells between pH 5 and 7 (*P* = 0.888), while no survival was observed at pH 2 or 3. The presence of up to 1.2% of bile in the media had no effect on *M. hypermegale* survival (*P* = 0.374). Oxygen exposure for 60 min reduced the number of viable cells of *M. hypermegale* by 41.7% (from 5.1 × 10^4^ to 3 × 10^4^ [*P* = 0.047]), but no effects on survival were observed after 15- and 30 min of oxygen exposure compared to initial bacterial abundance (time 0). Co-culture of *M. hypermegale* and *S*. Typhimurium reduced *Salmonella* counts by 97.7% (from 8.63 × 10^9^ to 2.0 × 10^8^ [*P *= 0.009]); however, no inhibition zone was observed using the agar slab method. Co-culture of *M. hypermegale* and *S*. Enteritidis reduced *Salmonella* counts by 71.1% (from 6.98 × 10^8^ to 2.02 × 10^8 ^[*P* = 0.003]) (Fig. S2; Table S4).

### *M. hypermegale* introduced twice as fresh culture efficiently colonized broiler ceca

**Fig 1:**
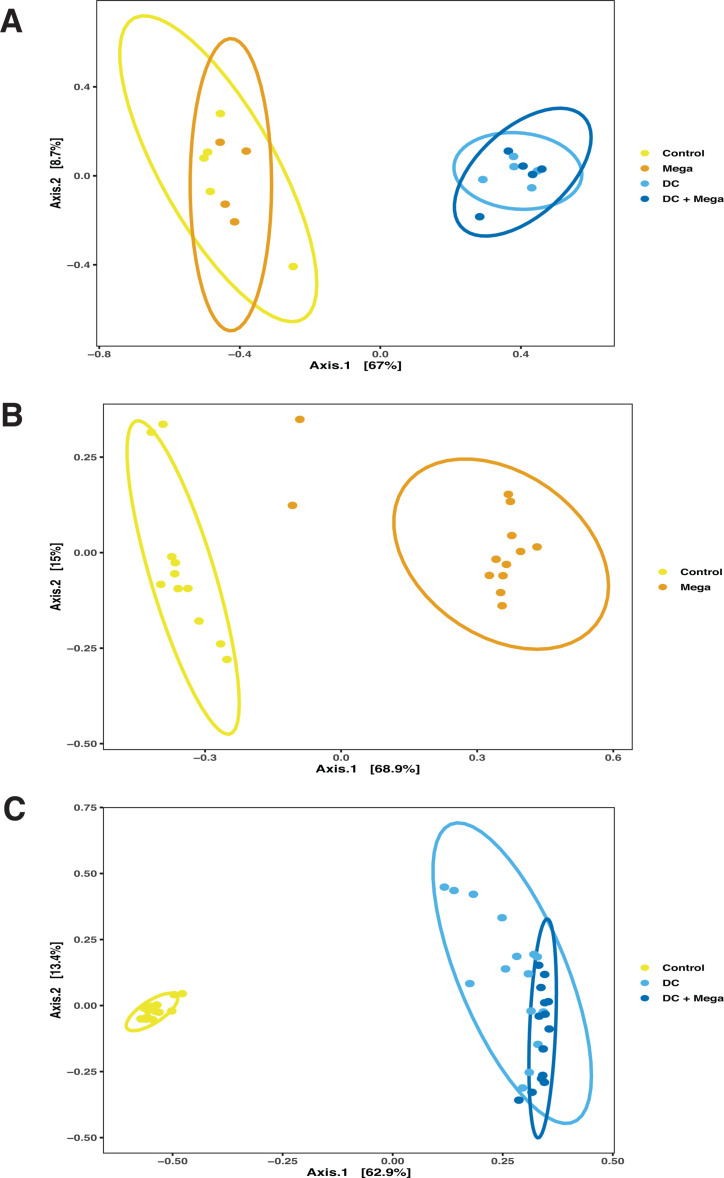
Effect of inocula on microbial beta-diversity. Principal coordinates analysis (PCoA) generated based on Bray-Curtis dissimilarity matrix of cecal samples obtained from 14-day-old Control chicks and chicks colonized with Mega, DC or DC + Mega treatments in EXP1 (**A**), EXP2 (**B**), and EXP3 (**C**). Samples are colored and shaped according to treatments, and data ellipses represent the 95% confidence region for group clusters assuming a multivariate t-distribution.

**Fig 2:**
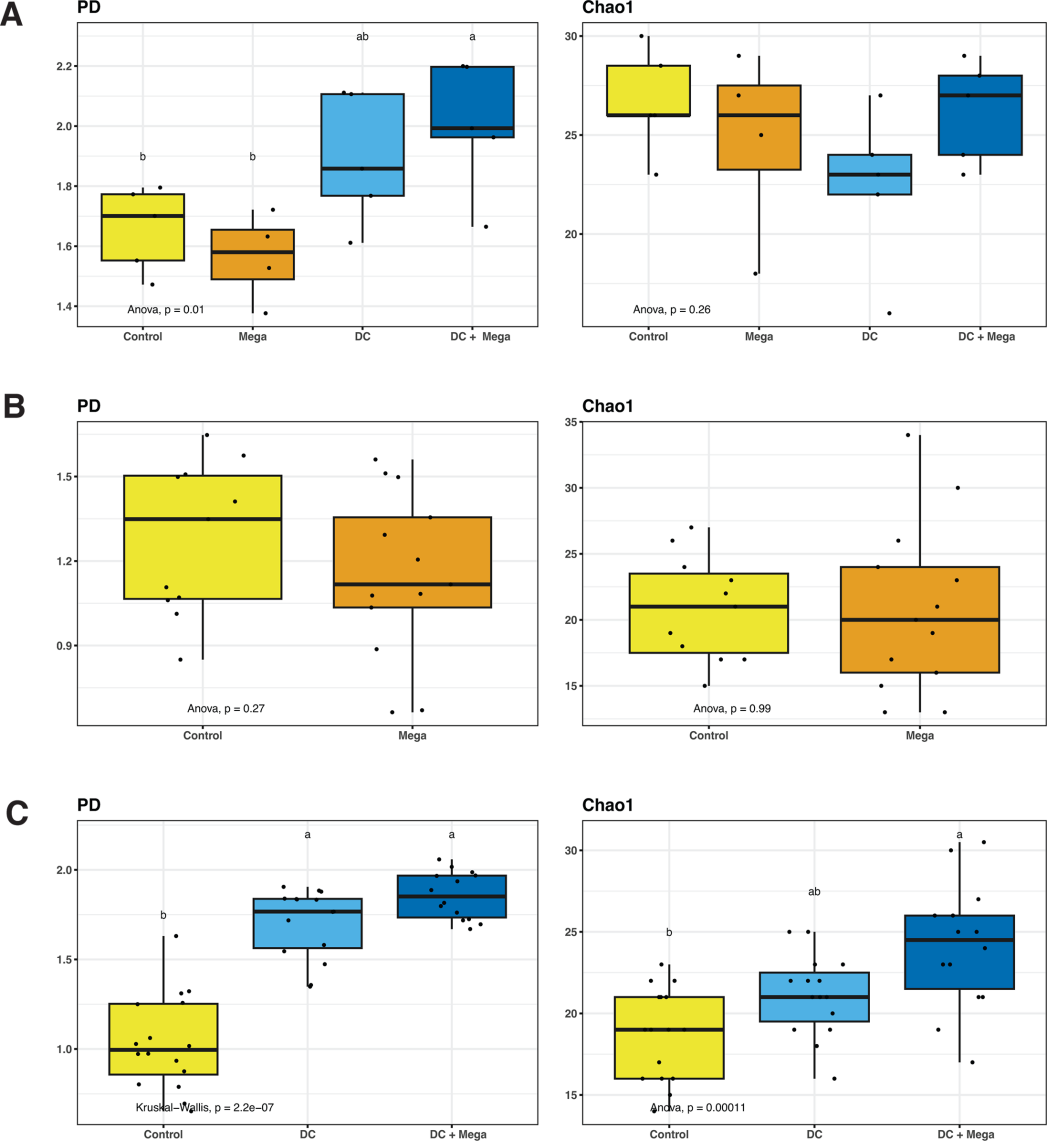
Alpha-diversity indices PD and Chao1 of cecal samples obtained from 14-day-old Control chicks and chicks colonized with Mega, DC or DC + Mega treatments in EXP1 (A), EXP2 (B), and (C) EXP3. Superscripts with different letters indicate significant differences at *α* = 0.05.

In EXP1, the *M. hypermegale* strain introduced from a frozen glycerol stock failed to colonize the chicken ceca and was not detected in the Mega or DC + Mega birds. At day 14, beta-diversity analysis indicated that the cecal microbiota of Control and Mega birds was different from that of DC and DC + Mega birds (*r*^2^ = 0.70, *P* = 0.001); however, no differences were found in the cecal microbiota between Control and Mega birds, nor between DC and DC + Mega birds (Fig. 1A). The DC + Mega birds presented higher PD than that of Control (*P* = 0.03) and Mega (*P* = 0.01) birds, but no differences were observed in Chao1 index (*P = *0.26) (Fig. 2A). In EXP2, *M. hypermegale* introduced twice from a fresh culture successfully colonized the chicken gut. Gut microbial structures, as indicated by beta-diversity matrix, were different between Control and Mega birds (*r*^2^ = 0.60, *P* = 0.001) (Fig. 1B). No differences in PD and Chao1 index (*P* = 0.355, *P = 0.96*, respectively) were found between Control and Mega birds (Fig. 2B). In EXP3, beta-diversity analysis indicated that the cecal microbiota of control group was different from that of DC and DC + Mega (*r*^2^ = 0.64, *P* = 0.001 and *r*^2^ = 0.71 and *P* = 0.001, respectively) and that DC and DC + Mega microbial communities also differed (*r*^2^ = 0.26, *P* = 0.001) (Fig. 1C). The microbiota of birds treated with DC or DC + Mega presented higher PD than the Control group (*P* < 0.001), and Chao1 index was higher in DC + Mega compared to Control group (*P* < 0.001) (Fig. 2C).

**Fig 3:**
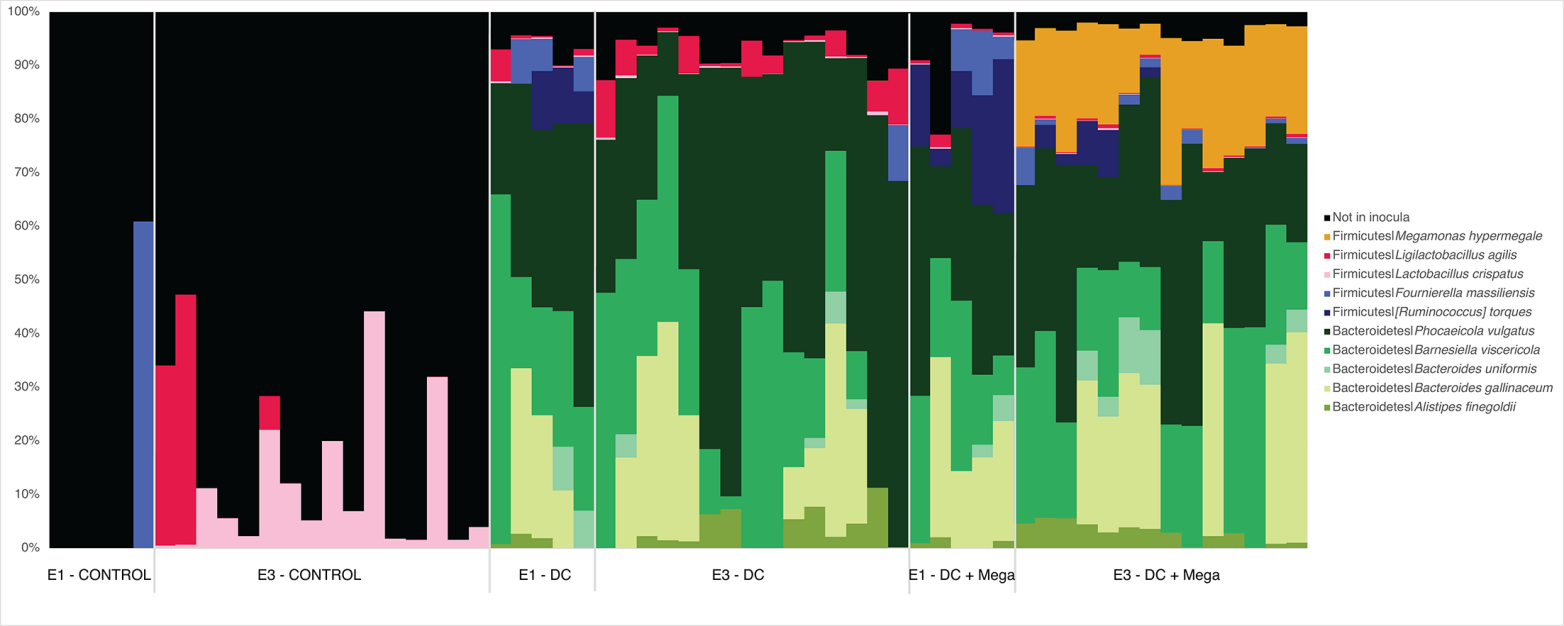
Bar plots of the relative abundances of bacterial species included in the DC and DC + Mega inocula detected in cecal samples obtained from 14-day-old chicks from control, DC, and DC + Mega treatments in EXP1 and EXP3. Species not included in the inocula were considered baseline microbiota and combined into as “Not in inocula” and shown in black.

**Fig 4:**
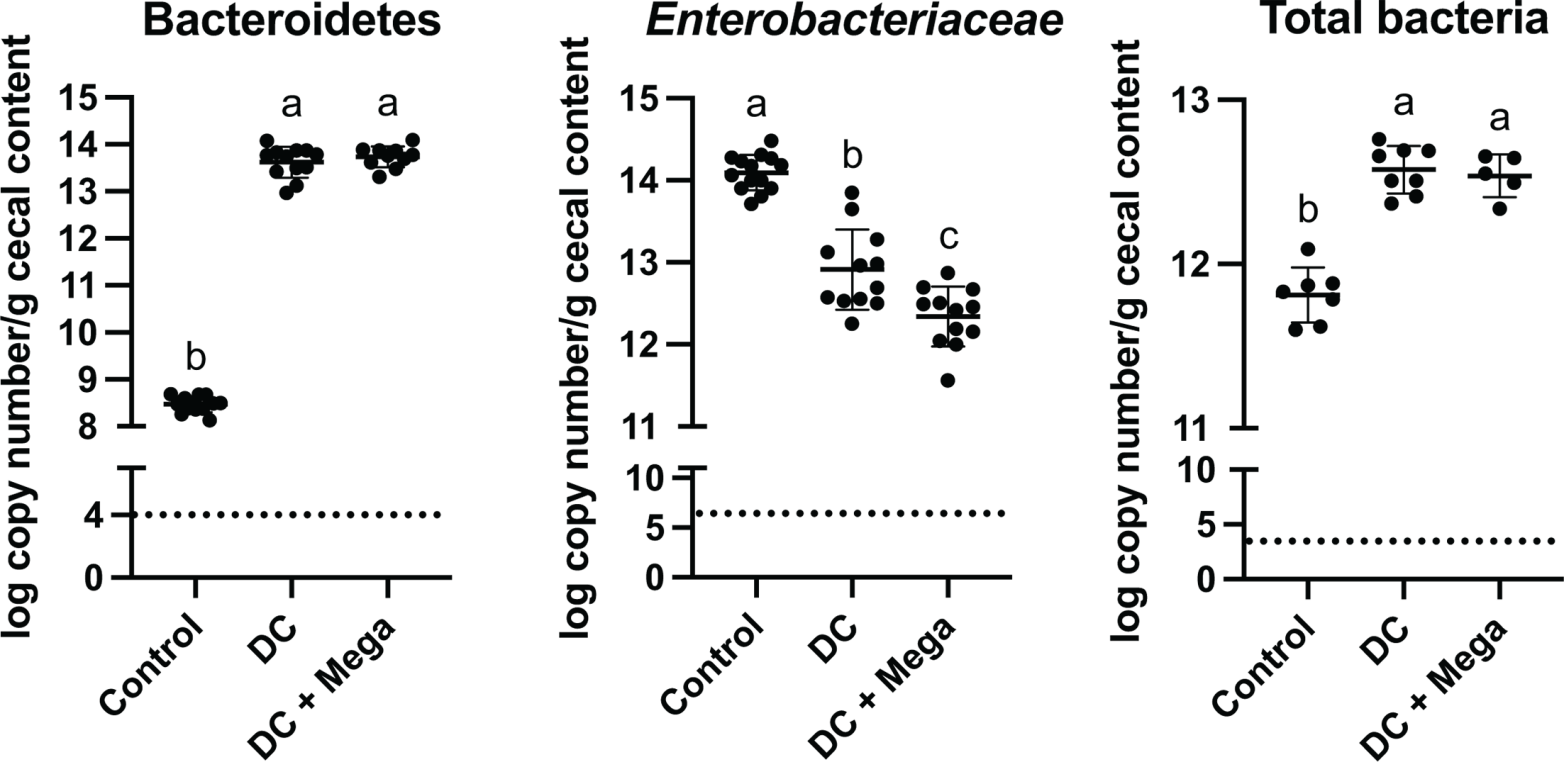
Effect of bacterial inoculation on Bacteroidetes, *Enterobacteriaceae,* and total bacteria load in ceca determined by qPCR. Superscripts with different letters indicate significant differences at *α* = 0.05. The dotted line represents the limit of detection according to a negative control (no DNA template).

In EXP1 and EXP3, an average of 94.0% ± 4.0% of the cecal microbial community of DC and DC + Mega groups were ASVs found in the DC inoculum, whereas only 15.2% ± 18.5% of the cecal microbial community in Control birds represented ASVs found in the DC inoculum. Bacteria shared between Control and DC inocula included: *F. massiliensis* detected in one control bird in EXP1 at 60.9% relative abundance; *L. agilis* detected in 3 out of 16 birds in EXP3 with average relative abundance of 28.8% ± 20.5%, and *L. crispatus*, which was consistently detected in all Control birds from EXP3, with average relative abundance of 10.7% ± 12.7%. Due to consistently being detected in all control birds, the ASV assigned to *L. crispatus* was considered a shared member of the baseline/initial microbiota in EXP3 (Fig. 3). In EXP3, the cecal microbiota of Control birds was dominated by Firmicutes (an average of 50.8% ± 12.8%) and Proteobacteria (an average of 49.2% ± 12.8%), whereas the cecal microbiota of DC and DC + Mega birds were dominated by Bacteroidetes (an average of 86.3% ± 7.1% and 73.1% ± 6.3%, respectively), Firmicutes (an average of 8.8% ± 5.8% and 24.8% ± 6.3%, respectively), and Proteobacteria (an average of 4.8% ± 2.5% and 2.0% ± 0.9%, respectively) made up a small proportion of the community. Consistently, qPCR results indicated birds in DC and DC + Mega treatments had a higher load of Bacteroidetes (*P* < 0.001) and total bacteria (*P* = 0.001), and a lower load of *Enterobacteriaceae* (*P* = 0.002) than the birds in control group (Fig. 4).

**Fig 5:**
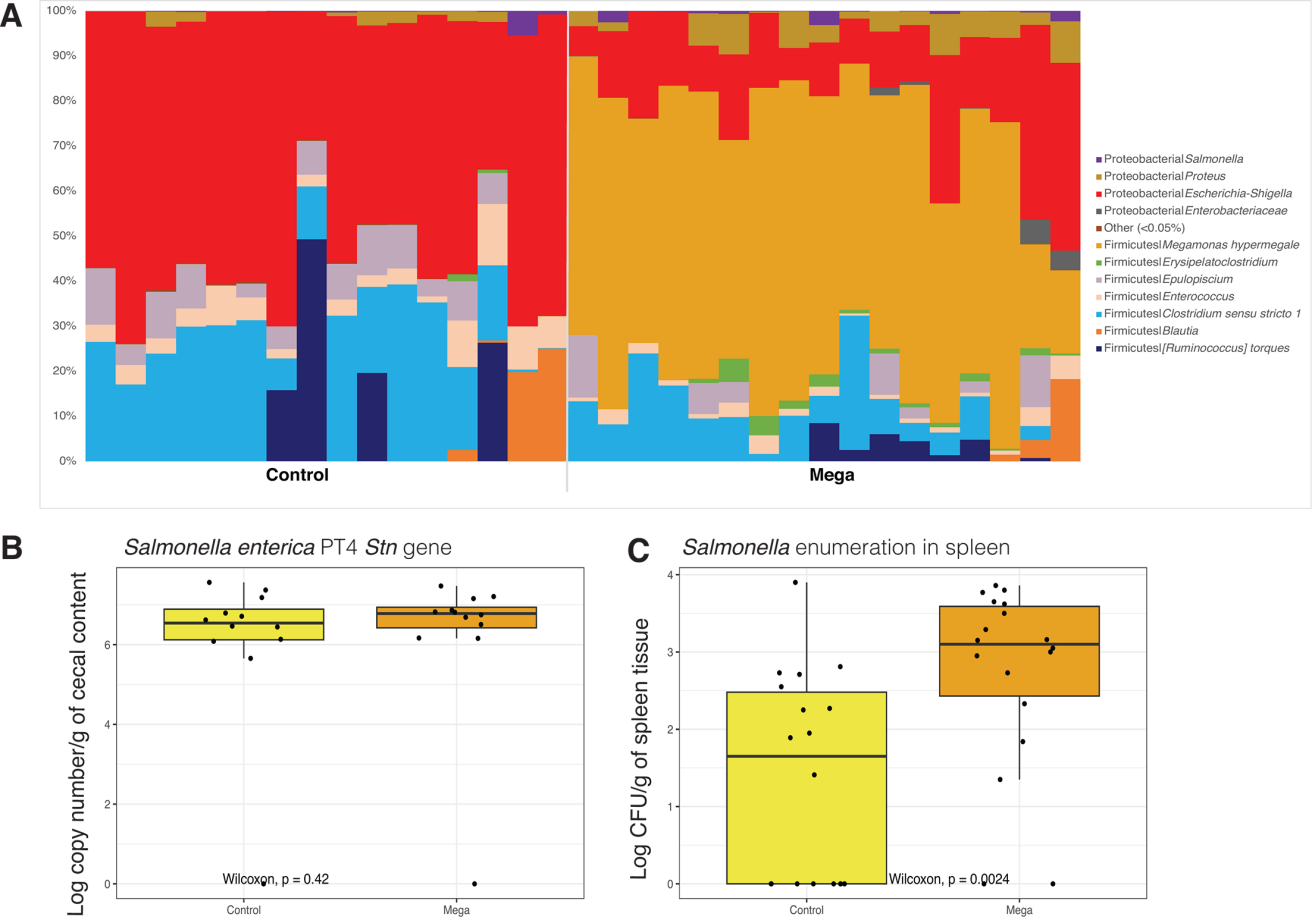
(**A**) Bar plots showing the relative abundances of bacterial species detected in cecal samples obtained from 14-day-old chicks from Control and Mega treatments in EXP2. Dendrograms showing the effect of treatments on *Salmonella* colonization in (**B**) cecal contents based on *Stn* gene quantification by qPCR and in (**C**) spleen tissues based on culturing and enumeration method.

In EXP2, *M. hypermegale* given as a fresh culture successfully colonized the ceca of treated birds and relative abundance ranged from 18% to 73%, with an average of 57.0% ± 15.9% (Fig. 5A). *M. hypermegale*-treated birds presented lower relative abundance of *Enterococcus* (*P* = 0.001) and *Escherichia-Shigella* than Control birds (*P* < 0.001). *Salmonella* enumeration of intestinal samples by plating was unsuccessful due to overgrowth on the plates by non-salmonella colonies; therefore, qPCR for *Salmonella* was completed. There were no differences in cecal *Salmonella* load as determined by qPCR (Fig. 5B); however, enumeration technique showed a higher *Salmonella* load in the spleen of Mega-treated birds (*P* = 0.002, Fig. 5C).

**Fig 6:**
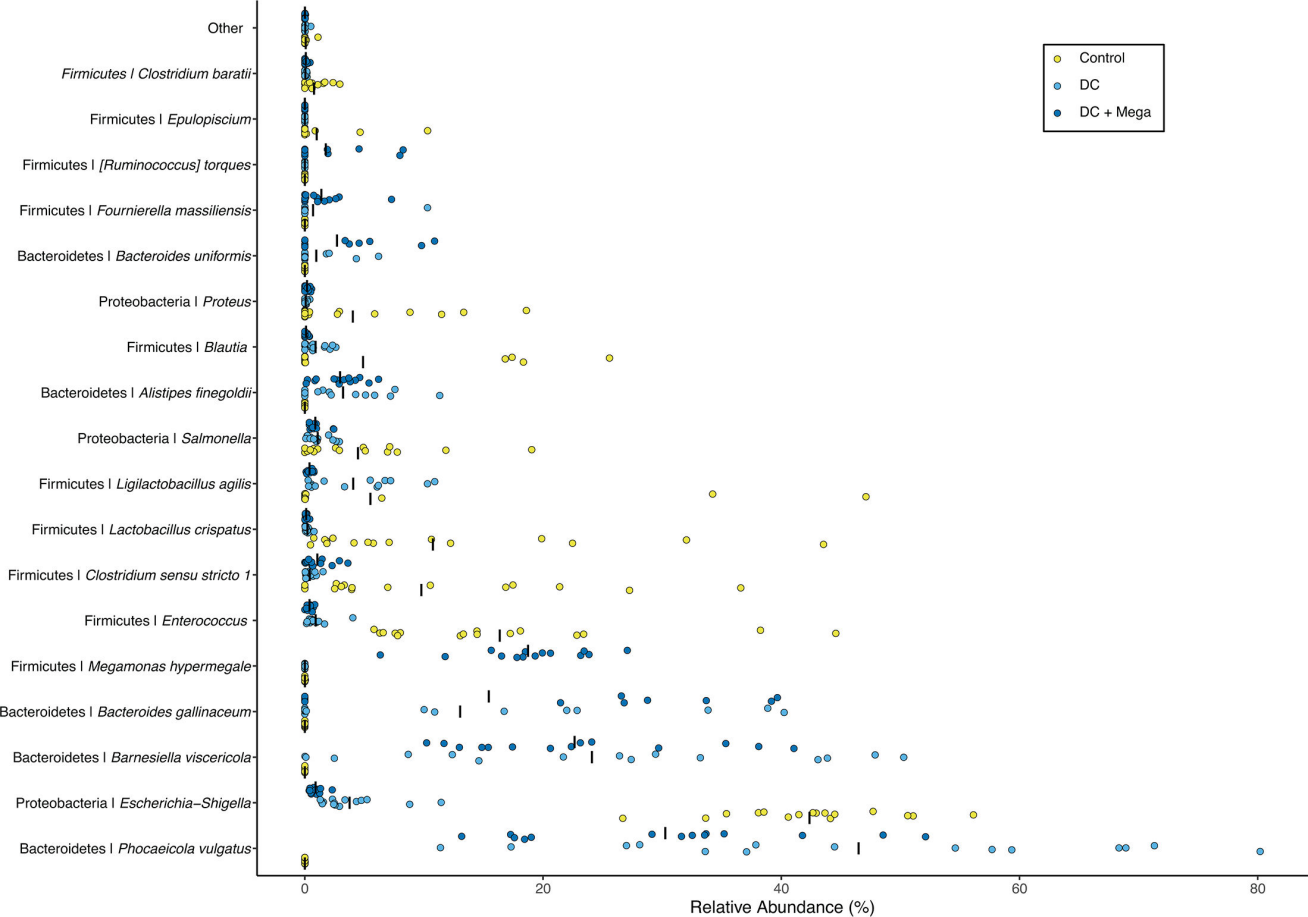
The relative abundance of microbial taxa that were shown to be differentially abundant in the cecal microbiota of 14-day-old broilers from Control, DC, and DC + Mega treatments in EXP3 according to DESeq2 analysis. Dots represent the relative abundance of taxa in individual samples, and bars represent the average relative abundance in each treatment. The relative abundance of taxa that were not differently abundant is shown as “Other.”

**Fig 7:**
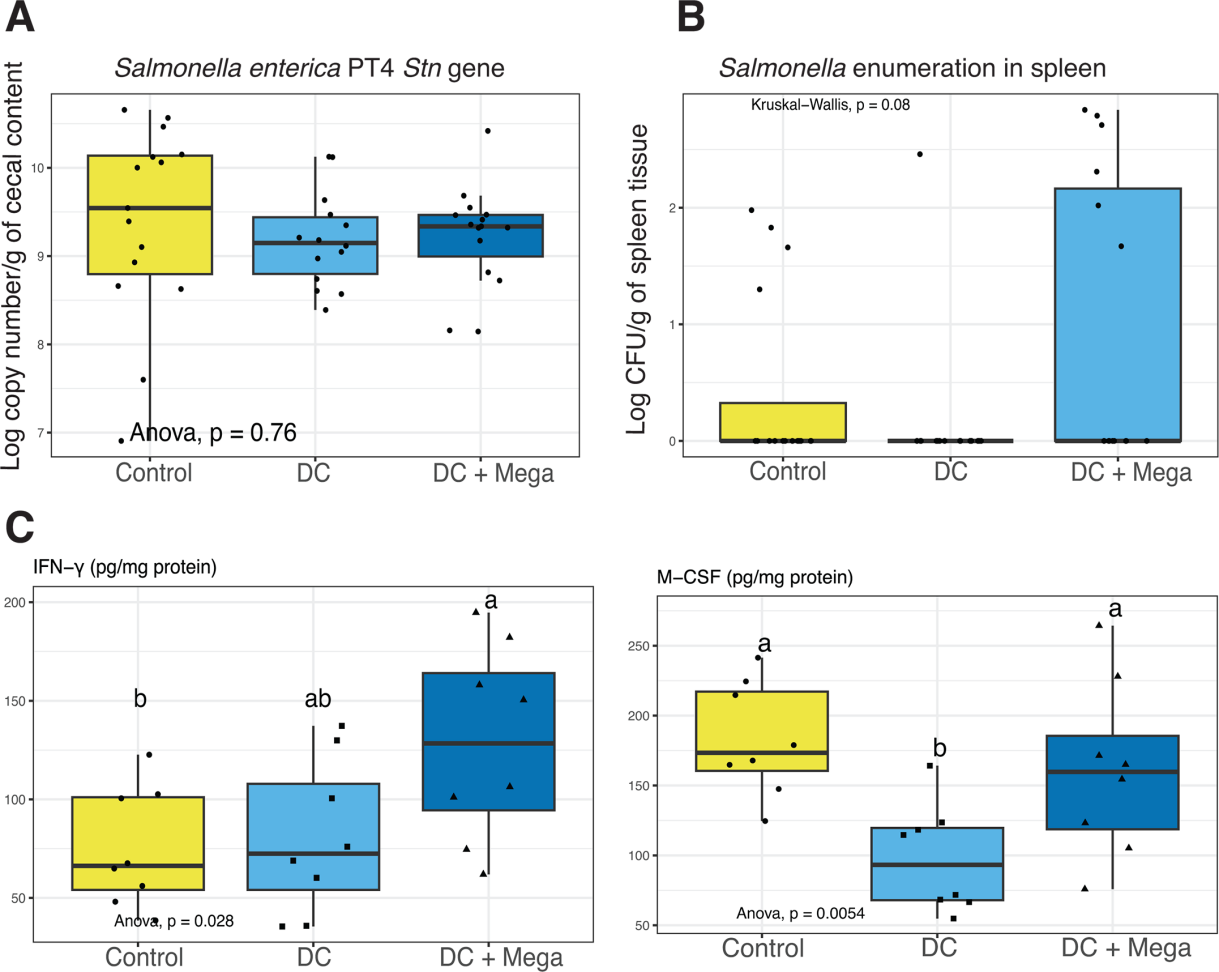
Boxplots showing the effect of Control, DC and DC + Mega treatments in EXP3 on *Salmonella* colonization in (**A**) cecal contents based on *Stn* gene quantification by qPCR and in (**B**) spleen tissues based on culturing and enumeration methods. (**C**) Boxplots showing the effects of treatments on the concentration of IFN-γ and M-CSF in the cecal tissues of 14-day-old broilers. Superscripts with different letters indicate significant differences at *α* = 0.05.

In EXP3, differential abundance analysis indicated that DC and DC + Mega birds presented higher relative abundance of inocula ASVs coinciding with *B. gallinaceum*, *B. uniformis*, *A. finegoldii*, *P. vulgatus*, and *B. viscericola*, and lower relative abundance of *Escherichia-Shigella*, *Proteus*, *Enterococcus*, *Clostridium sensu stricto 1*, and *L. crispatus* (*P* < 0.001) than birds in the Control group. In addition, DC + Mega birds showed a lower relative abundance of *Salmonella* (*P =* 0.006), and higher levels of inocula ASVs assigned to *F. massiliensis*, *R. torques*, and *M. hypermegale* (*P <* 0.001) than Control birds (Fig. 6). Comparisons between DC and DC + Mega treatments indicated DC cecal microbiota to be enriched for *Escherichia-Shigella* and *L. agilis*, whereas the cecal microbiota of DC + Mega showed enriched *M. hypermegale*, *R. torques*, *F. massiliensis* (*P* < 0.001) and *Clostridium sensu stricto 1* (*P* = 0.02) (Fig. S3). While 16S sequencing results indicated a reduction in *Salmonella* load in DC + Mega treated birds compared to control group (*P* = 0.006), no differences in *Salmonella* load were observed using qPCR (*P* = 0.76). However, the mean log_10_ copy number/g of cecal content of *stn* gene was numerically lower in DC and DC + Mega treated birds compared to control (Fig. 7A). *Salmonella* load in the spleen was not different (*P* = 0.08) with *Salmonella* being undetectable in most samples (Fig. 7B). Correlation analysis performed between taxa present in the inocula and the baseline microbiota indicated *M. hypermegale* to be negatively correlated with *Escherichia*/*Shigella*, whereas *Salmonella* was negatively correlated with *B. viscericola* and positively associated with *P. vulgatus* and *A. finegoldii* (Fig. S4).

**Fig 8:**
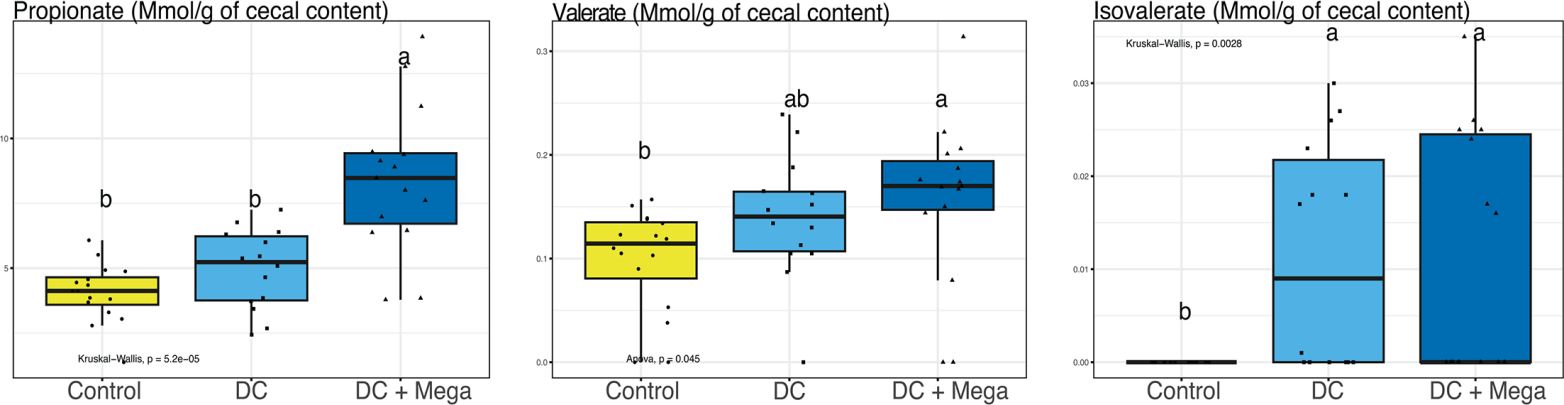
Boxplots showing the effect of Control, DC, and DC + Mega treatments in EXP3 on the concentration of short-chain fatty acids in the cecal content of 14-day-old broilers. Superscripts with different letters indicate significant differences at *α* = 0.05.

Birds in the DC + Mega treatment showed a higher level of IFN-γ (*P* = 0.028) in cecal tissue than birds in the Control group. Cecal tissue of birds in the DC treatment exhibited the lowest M-CSF concentration among treatment groups (*P* = 0.005) (Fig. 7C), which may coincide with the lower relative abundance of *Salmonella* and the numerical pattern of lower spleen translocation in this group (*P* = 0.108). Cecal concentration of isovalerate was higher in birds from DC and DC + Mega treatments compared with Control (*P* = 0.002) Valerate concentration was higher in DC + Mega compared to Control birds (*P* = 0.045). Propionate (*P* < 0.001) concentration was higher in DC + Mega treated birds compared with birds in Control and DC treatments (Fig. 8).

### *B. viscericola* and *P. vulgatus* strains effectively colonize the chicken ceca

**Fig 9:**
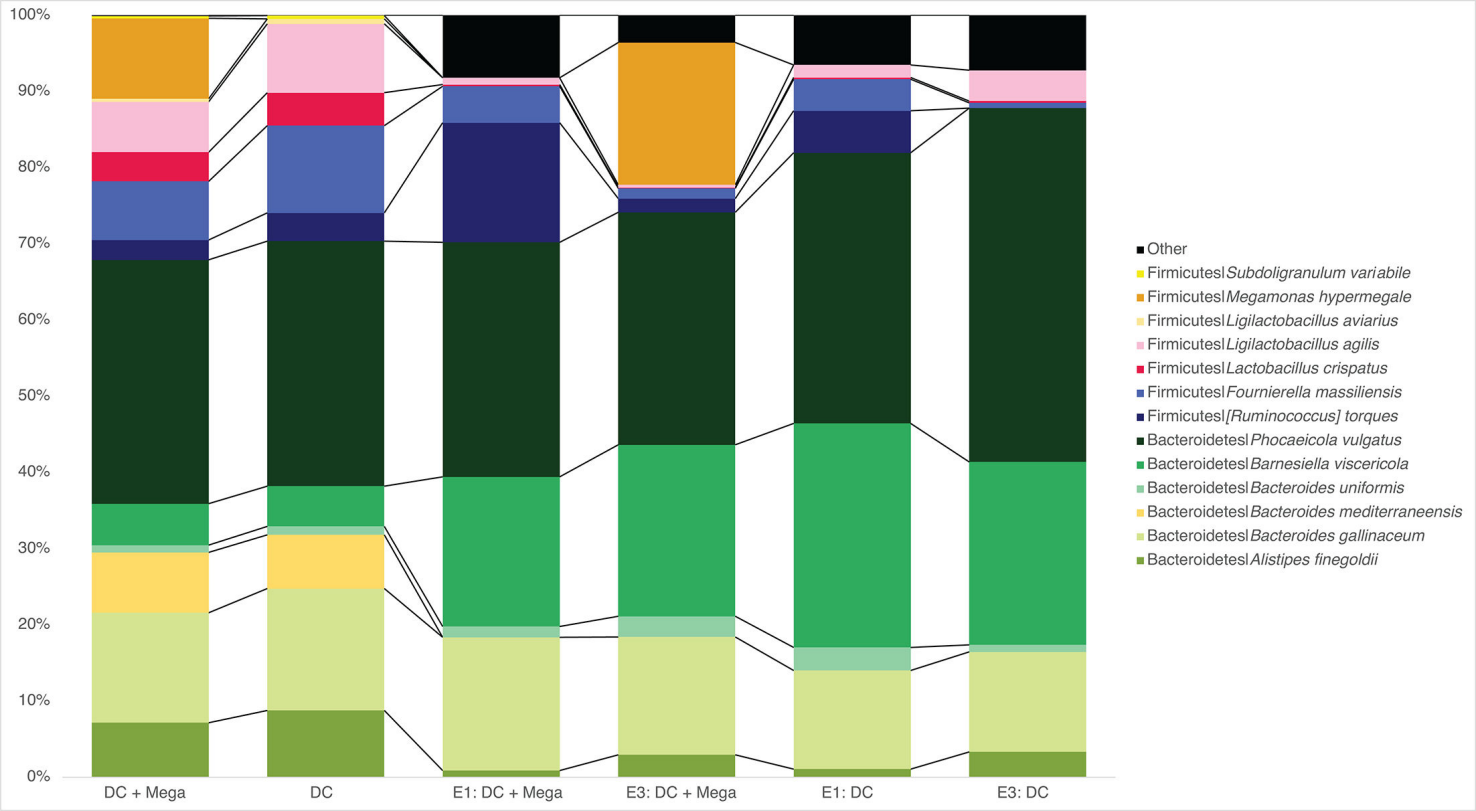
Barplots showing the relative abundance of species included in the DC + Mega and DC inocula and the average relative abundance of these species in the inoculated birds in EXP1 and EXP3. Species present in the birds but not introduced by the inocula were combined as “Other” and are shown in black.

Colonization ability of strains included in the inocula was determined based on the prevalence of isolates in the ceca of inoculated birds. These were evaluated at ASV level, and results are shown at the taxon level. *A. finegoldii*, *B. gallinaceum*, *B. viscericola*, *P. vulgatus*, and *L. agilis* strains were detected in more than half of the inoculated birds and demonstrated high colonization ability. Particularly, *B. viscericola*, *P. vulgatus,* and *L. agilis* strains were detected in all birds. *M. hypermegale* isolate failed to colonize the ceca when introduced from a frozen glycerol stock in EXP1 but colonized all the birds and presented a high abundance when introduced as fresh culture in two inoculations in EXP2 and EXP3. The *B. mediterraneensis*, *L. aviarius*, and *S. variabile* strains included in the DC consistently failed to colonize the chicken gut (Fig. 9).

## DISCUSSION

In this study, a DC containing 12 bacterial isolates harvested from adult chicken ceca was inoculated to young chicks, resulting in increased phylogenetic diversity and substantial changes in the cecal microbiota composition evaluated at 14 days old in the context of a *Salmonella* challenge. Strikingly, Bacteroidetes phylum made up more than 75% of the cecal microbiota of DC-inoculated birds but were undetectable in Control birds. Bacteroidetes species were reported to be host-adapted (23) and *Alistipes*, *Bacteroides*, *Barnesiella*, and *Phocaeicola* genera were found to be good colonizers of the chicken ceca, either when introduced as isolates or as part of complex and defined communities (11, 23, 51, 52). Bacteroidetes are non-spore forming and sensitive to oxygen; therefore, they have low ability to survive in the environment and are likely to be lost, reduced, or to colonize with delay in broilers due to practices employed in the poultry industry that hinder the contact between newly hatched chicks and their parental microbiota (3, 53). Consequently, it is likely that, once Bacteroidetes are introduced to the gut environment, they can occupy available niches and efficiently engraft.

Despite the substantial differences in cecal microbiota composition, the differences in host responses measured in DC-inoculated and Control birds were subtle. Changes were observed in IFN-γ, M-CSF, propionate, isovalerate, and valerate concentration in the ceca, but no significant differences were found in body weight, small intestine morphology, and other cytokines measured. Similarly, a previous study found that the inoculation of nine bacteria obtained from chickens greatly affected the chick gut microbiota composition but caused only a transient increase in systemic IgA levels (54).

Birds colonized with DC + Mega had higher concentrations of valerate and isovalerate compared to control birds and higher concentration of propionate compared to both Control and DC inoculated birds. Consistent with the current study, we previously found that the concentrations of valerate and propionate were higher in cecal contents of chicks inoculated with microbial cultures and cecal contents from adult birds (9). This suggests that inoculation with the DC community partially recapitulated the effects of introducing a complex microbial community regarding the production of SCFAs, with the introduced *M. hypermegale* strain likely playing a major role in propionate production. As seen in other studies, the *M. hypermegale* strain used in our study is a propionate producer and metabolizes free hydrogen, which can favor the production of SCFAs by other members of the community (18, 55). Although the introduction of DC or DC + Mega were shown to reduce the relative abundance of *Salmonella* in the cecal contents as indicated by 16S rRNA sequencing, only a numeric decrease in *stn* gene abundance was observed using qPCR analysis.

We speculated two main reasons why the inoculation with DC did not significantly impact *Salmonella* cecal load compared with Control birds. First, the DC we inoculated had a limited number of microorganisms. Significant reductions in *Salmonella* load were usually observed in studies inoculating defined communities containing 25 species or more (19, 56, 57), or using commercial products (58) from which the bacterial composition is not disclosed but is likely to include diverse species. As an exception, a study found that inoculation of germ-free chicks with 10 bacterial isolates harvested from the ceca of feral chickens resulted in a significant reduction in *Salmonella* load and ameliorated intestinal inflammation (59). In that way, rather than a low number of microorganisms, it is possible that the microorganisms included in our study may not be highly effective to promote *Salmonella* resistance. A second possible explanation is the fact that the baseline microbiota of our birds was enriched with *Escherichia/Shigella,* which made up an average of 42.0% ± 12.0% of the cecal microbial community in Control birds; however, in birds inoculated with DC and DC + Mega, the average relative abundance of *Escherichia/Shigella* was less than 5%. It is possible that the high abundance of *Escherichia/Shigella* within the ceca of Control birds promoted a similar level of protection of hosts to *Salmonella* infection to that of the DC or DC + Mega treatment. Commensal *Enterobacteriaceae* species were previously demonstrated to protect chickens against *Salmonella* infection through oxygen competition (60). The proposed mechanism indicates that, in the presence of inflammatory signaling, butyrate produced by clostridia stimulates peroxisome proliferator-activated receptor gamma signaling pathway that results in mitochondrial oxidation and epithelial hypoxia (60). The maintenance of epithelial hypoxia by clostridia and the consumption of oxygen by *Enterobacteriaceae* hinder the ability of *Salmonella* to perform aerobic respiration with decreased colonization ability (60). In the present study, *Clostridium Stricto Sensu 1* made up an average of 16.1% of the microbial community in the Control birds but had lower relative abundance in DC and DC + Mega groups (0.8% and 2.3%, respectively). Despite the absence of differences in *Salmonella* load quantified by qPCR, the fact that DC and DC + Mega reduced the relative abundance of *Escherichia/Shigella* and *Salmonella*, as well as reduced *Enterobacteriaceae* load could be interpretated as a positive outcome. Currently, avian pathogenic *Escherichia coli* is one leading cause of antibiotic treatment in broiler flocks in Canada (61), and the use of DC could be investigated as a strategy to mitigate the occurrence of *Escherichia/Shigella-*associated diseases.

In EXP1, *M. hypermegale* provided from a frozen glycerol stock failed to colonize the birds. This was largely unexpected, as *Megamonas* has been shown to be a good colonizer in birds inoculated with frozen cecal contents, cecal cultures, and competitive exclusion products (9, 62). Even if accidentally exposed to oxygen during the inoculation procedure, the *M. hypermegale* strain used in our study was shown to survive for at least 30 min. In EXP2, birds were inoculated with fresh *M. hypermegale* broth, resulting in successful colonization; however, birds in Mega treatment had higher *Salmonella* counts in the spleen than Control birds, indicating that introduction of *M. hypermegale* alone, without an accompanying DC, might be detrimental.

The *in vitro* acid tolerance assay showed *M. hypermegale* did not survive at pH 3 and lower. Although the pH of gizzard and proventriculus ranges from 2 to 5.5, the presence of digesta buffers acidity and provide nutrients, such as glucose, which can improve bacterial survival (63). These conditions may allow *M. hypermegale* to colonize as it presents sufficient resistance to acidic conditions *in vivo*. The two *in vitro* inhibition assays targeting *Salmonella* Typhimurium resulted in different outcomes: while co-culture assay indicated inhibitory effect of *Salmonella* by *M. hypermegale*, the same was not observed using the slab method. These phenotypes might be explained by differences in metabolism of bacteria when growing in solid or liquid media (64) and optimization of *in vitro* methods is necessary to better understand such differences.

Overall, the results indicated that inoculation with DC and *M. hypermegale* can cause significant shifts in microbiota composition without major effects on broiler physiology or the ability to inhibit *Salmonella* colonization. Among the limitations of this study, the load of each species included in the DC was not quantified before inoculation. Thus, it is possible that *B. mediterraneensis*, *L. aviarius*, and *S. variabile*, which consistently failed to colonize the birds, were not provided at sufficient load to promote colonization. On the other hand, it is possible that strains deemed efficient colonizers were favored by being provided at higher loads in our study. Future studies are warranted using individual isolates introduced at comparable concentration for fair comparison of colonization ability. In addition, a comparison between the introduction of isolates using fresh and frozen cultures is needed.

The DC used in this study was designed aiming to promote the establishment of a microbiota community resembling that of a typical broiler and included the same number of species from Bacteroidetes and Firmicutes phyla (six each) because major differences in the relative abundances of these phyla were observed between intensive and extensive rearing systems (2). Proteobacteria species were not included in the DC as they are highly abundant in the microbiota of newly hatched chicks. According to previous data, a typical broiler microbiota would present a relative abundance of Bacteroidetes and Firmicutes ranging from 20% to 65% and that of Proteobacteria ranging from 2% to 10%. Despite the presence of Firmicutes in the DC and of Proteobacteria in the baseline community, the microbiota of DC-treated birds was dominated by Bacteroidetes, reaching up to 96% in some individuals, which could be reflective of an unbalanced microbial composition. Therefore, future studies using different combinations of isolates at known concentrations are warranted to better understand microbiota composition changes in chicks exposed to DC and to support the development of gnotobiotic models harboring typical broiler microbiota, enabling the evaluation of functional roles of individual microbial species and selection of bacterial strains that can benefit poultry health and production.

### Conclusion

We concluded that the introduction of a DC containing 12 bacterial strains to day-old chicks caused major changes in cecal microbiota composition with subtle effects on host physiology and the ability to resist *Salmonella* challenge. We identified* A. finegoldii*, *B. gallinaceum*, *B. viscericola*, *P. vulgatus,* and *L. agilis* strains used in this study as effective colonizers of the chicken gut and found that the introduction of the DC causes significant reduction in the relative abundance of *Escherichia-Shigella* and *Salmonella*, as well as *Enterobacteriaceae* load, which could potentially be explored as a strategy to control the occurrence of *Escherichia/Shigella*-associated diseases.

## Data Availability

The 16S rRNA sequencing data generated and analyzed in the current study are available in the NCBI SRA repository under accession no. PRJNA1144928.

## SUPPLEMENTAL MATERIAL

Supplemental figures aem.00948-25-s0001.docx Fig. S1 to S4.

Table S1 aem.00948-25-s0002.xlsx Community characteristics.

Supplemental tables aem.00948-25-s0003.docx Body weight and histology data.

Table S4 aem.00948-25-s0004.xlsx *In vitro* experiments.

ASM does not own the copyrights to Supplemental Material that may be linked to, or accessed through, an article. The authors have granted ASM a non-exclusive, world-wide license to publish the Supplemental Material files. Please contact the corresponding author directly for reuse.
