# Supplementary material for: Impact of a defined bacterial community including and excluding Megamonas hypermegale on broiler cecal microbiota and resistance to Salmonella infection
Source: Appl Environ Microbiol. 2025 Aug 19;91(9):e00948-25. doi: 10.1128/aem.00948-25 (PMC12442376; doi:10.1128/aem.00948-25)
Supplement: Supplemental figures — Fig. S1 to S4. [file aem.00948-25-s0001.docx]

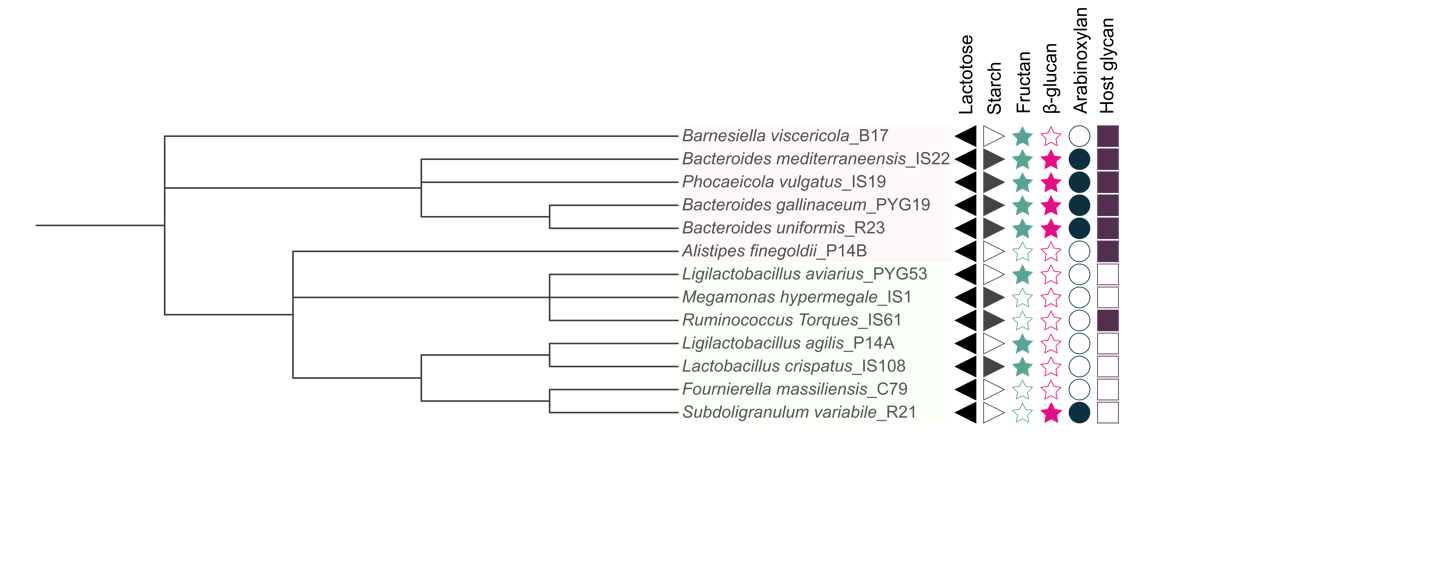


**Supplemental Figure 1.** Carbohydrate utilization capability of isolates included in the defined community as determined by alignment of 16S rRNA gene sequences against Carbohydrate Active Enzymes Database (CAZyme). Solid shapes represent the presence of enzymes to degrade listed substrates.


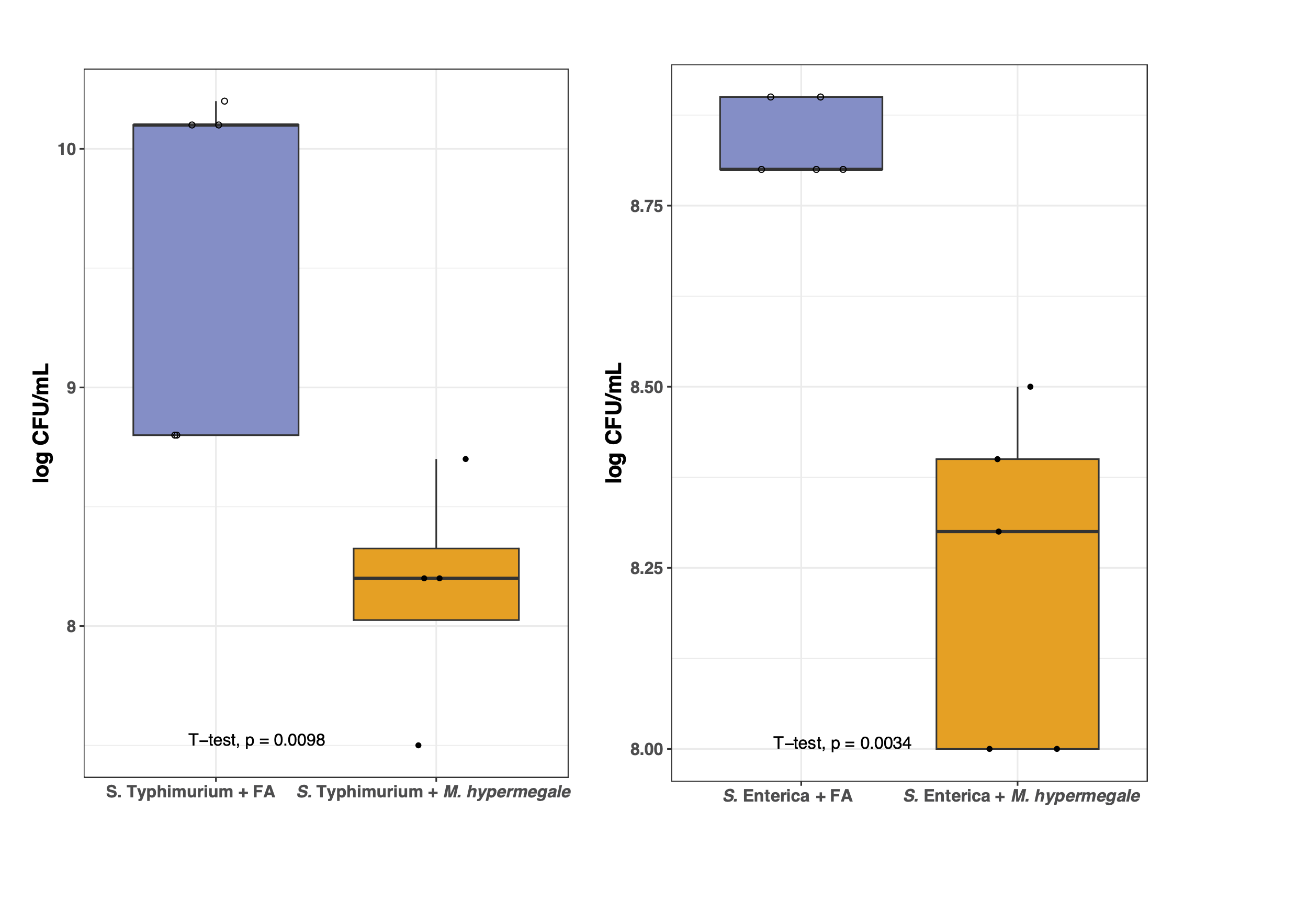


**Supplemental Figure 2.** Bacterial enumeration of 24 h cultures of *Salmonella* in fastidious anaerobe (FA) broth and co-cultures of *Salmonella* and *Megamonas hypermegale*.

**Supplemental Figure 3.** Microbial taxa that were shown to be differentially abundant in the cecal microbiota of 14-day-old broilers from DC (light blue, negative values) and DC + Mega treatments (dark blue, positive values) in EXP3 according to DESeq2 analysis.

**Supplemental Figure 4.** Heatmap showing Spearman correlation between bacterial taxa present in the inocula (rows) and the baseline microbiota (columns) for birds inoculated with DC in EXP3. Asterisks denotes significant associations p-values of <0.05 (*) and <0.01 (**).
