# Supplementary material for: Impact of a defined bacterial community including and excluding Megamonas hypermegale on broiler cecal microbiota and resistance to Salmonella infection
Source: Appl Environ Microbiol. 2025 Aug 19;91(9):e00948-25. doi: 10.1128/aem.00948-25 (PMC12442376; doi:10.1128/aem.00948-25)
Supplement: Supplemental tables — Body weight and histology data. [file aem.00948-25-s0003.docx]

**Supplemental Table 2**. Body weight (average ± SD, g) in all treatments across experiments

| **Treatment** | **Control** | **Mega** | **DC** | **DC + Mega** |
| --- | --- | --- | --- | --- |
| **14-days-old** |  |  |  |  |
| EXP1 | 409.5 ± 47.1 | 430.2 ± 61.5 | 415.7 ± 47.3 | 419.3 ± 48.7 |
| EXP2 | 413.5 ± 52.2 | 408.9 ± 61.8 | NA | NA |
| EXP3 | 367.5 ± 32.2 | NA | 368.1 ± 51.4 | 363.6 ± 49.2 |
| **7-days-old** |  |  |  |  |
| EXP1 | 144.9 ± 14.1 | 153.9 ± 13.9 | 152.1 ± 18.3 | 154.7 ± 19.9 |
| EXP2 | 177.7 ± 25.5 | 183 ± 23.4 | NA | NA |
| EXP3 | 137.6 ± 11.0 | NA | 139.9 ± 51.4 | 138.6 ± 14.8 |
| **1-day-old** |  |  |  |  |
| EXP1 | 48.6 ± 4.0 | 48.4 ± 5.9 | 48.5 ± 3.5 | 48.4 ± 5.6 |
| EXP2 | 45.6 ± 3.3 | 46.0 ± 3.2 |  |  |
| EXP3 | 47.4 ± 3.1 | NA | 47.2 ± 3.0 | 47.7 ± 3.3 |

**Supplemental Table 3**. Effect of control, DC, and DC + Mega treatments on ileum morphology of 14-day-old broilers in experiment 3^a^.

|  | VH | SEM | CD | SEM | VW | SEM | VH/CD | SEM |
| --- | --- | --- | --- | --- | --- | --- | --- | --- |
| Control | 599.42 | 13.34 | 114.82 | 3.60 | 81.47 | 2.83 | 5.53 | 0.18 |
| DC | 665.64 | 18.68 | 128.05 | 4.41 | 85.42 | 3.42 | 5.47 | 0.21 |
| DC + Mega | 628.15 | 10.21 | 121.90 | 4.47 | 82.43 | 2.90 | 5.50 | 0.20 |
| *p*-value^b^ | 0.095 |  | 0.72 |  | 0.15 |  | 0.91 |  |

^a^VH, mean villus height (μm), CD, mean crypt depth (μm); VW, mean villus width (μm), VH/CD, mean villus height to crypt depth ratio; SEM, standard error of the mean.

^b^ Means were compared using Kruskal-Wallis test.
